# Miscellaneous Medical Intelligence

**Published:** 1850-07

**Authors:** 


### ARTICLE VI.

### MISCELLANEOUS MEDICAL INTELLIGENCE.

*Rush Medical College.*---The circular for the next course of lectures in this Institution, and the catologue of last session will be published in about two weeks.

The chair of Practice of Medicine in the University of Pennsylvania, made vacant by the resignation of Prof. Chapman has been filled by the transfer of Dr. George B. Wood to it.

The committee of the American Medical Association, appointed under Dr. Stille's resolution to offer a prize of $100 for the best experimental essay on a subject connected with physiology or medical chemisty, have announced that they will be ready to receive competing memoirs until the first day of March next, directed to Dr. F. G. Smith, Philadelphia.

The Supreme bench of Massachusetts have decided against the application for a new trial in the case of Prof. Webster.

